# Intravascular Lymphoma Presenting as Multiple Endocrine Failure, Transforming Into the Hemophagocytic Variant, and Relapsing as a Thrombotic Microangiopathy

**DOI:** 10.1155/crh/5560781

**Published:** 2025-09-17

**Authors:** Nigel P. Murray, Cinthia Escobar, Enrique Ramos, Claudia Perez, Dan Hartman

**Affiliations:** ^1^Department of Medicine, Hospital de Carabineros de Chile, Santiago, Chile; ^2^Faculty of Medicine, University Finis Terrae, Santiago, Chile; ^3^Department of Anatomy and Pathology, Hospital de Carabineros de Chile, Santiago, Chile

## Abstract

Intravascular large B-cell lymphoma (IVLCBL) is a rare form of non-Hodgkin's lymphoma and is characterized by the growth of large B-cells within blood vessels and bone marrow sinusoids. A 55-year-old man presented with a multiple endocrine failure which progressed to a pancytopenia. A bone marrow biopsy revealed a diffuse infiltration by large B-cells in the sinusoids consistent with intravascular lymphoma. After 6 cycles of R-CHOP, complete remission was achieved. Six months later, the patient relapsed presenting with a thrombotic microangiopathy which progressed to multiple organ failure and death.

## 1. Introduction

Intravascular large B-cell lymphoma (IVLCBL) is a rare form of non-Hodgkin's lymphoma characterized by the predominant growth of large cells within the lumen of blood vessels of differing sizes [1]. Although the localization of the lymphoma cells is within blood vessels, these cells are rarely detected in the peripheral blood, being absent in 90%–95% of cases [2]. The clinical presentation is variable ranging from monosymptomatic to paucisymptomatic forms, such as pyrexia, pain, or organ-specific local symptoms to a combination of B symptoms and multi-organ failure. Three variants have been described, the classical, with neurological symptoms in 35% and including multi-endocrine failure and pulmonary infiltrates, the cutaneous variant which presents with skin lesions, and finally the hemophagocytic syndrome–associated variant, presenting with bone marrow failure due to infiltration [3].

We present the case of a 55-year-old man who presented with multi-endocrine failure and weight loss and during a two-week clinical workup developed pancytopenia with a bone marrow biopsy consistent with intravascular lymphoma. Although achieving a complete remission, the patient relapsed and eventually died.

## 2. Clinical Case

A 55-year-old man presented to the Casualty Department at the end of October 2022 with a history of 5 months of increasing tiredness, loss of energy, loss of libido, and thoracic pain, worse on inspiration but without a cough. He also had noted a weight loss of approximately 25 kg over the last year although he had been dieting. His past medical history included hypertension, dyslipidemia, and resistance to insulin. His medication included valsartan, metformin, and atorvastatin. He had had a routine checkup in September 2022; the full blood count, biochemical and hepatic profile, and renal function were normal, but the free serum thyroxine (fT4) was low at 0.5 ng/dL (normal range 0.90–1.75 ng/dL) and the thyroid-stimulating hormone (TSH) was in the normal range (3.74 IU/mL) (normal range 1.85–3.85 IU/mL). Presenting to the Emergency Department in November 2022 with the aforementioned symptoms, the patient's blood tests revealed multiple endocrine failure together with an anemia, thrombocytopenia, and an elevated lactate dehydrogenase. A hypothesis of pituitary was made but a magnetic resonance of the pituitary fossa was informed as normal. The results of the hormone tests pretreatment and when in remission are shown in Table 1.

The full blood count in November of 2022 showed an anemia of 11.4 gr/dL, a MCV of 78 fL, a normal white cell count, and a platelet count of 97,000/mm^3^. The lactate dehydrogenase (LDH) was elevated at 858 U/L (normal range 0–232 U/L) with an elevated ESR of 90 mm/hr, a C-reactive protein of 122 mg/L (normal range < 5 mg/L), and a D-dimer of 1116 (normal range < 250).

A thoracic angio-computerized tomography showed a subsegmental pulmonary filling defect of the left posterior zone of the lung fields, suggestive of a pulmonary embolism, and as such was treated with dalteparin in therapeutic doses. It also showed the presence of hilar lymph nodes of < 10 mm and splenomegaly of 19 cm; there was no evidence of other adenopathies. Liver structure was normal with no evidence of cirrhosis or evidence of portal thrombosis. He was started on dalteparin and L-thyroxine and admitted for further studies.

He was treated in the first instance with L-thyroxine, hydrocortisone, and testosterone. Although feeling better with hormone replacement, his full blood count continued to deteriorate. Seven days after admission, a leukoerythroblastic anemia was detected with an increasing LDH (Table 2).

Although the patient had a low-grade fever, an infective cause was not detected. The patient's renal function started to deteriorate with proteinuria of 2.7 gr/24 h; serum light chain analysis showed a free kappa of 32.2 mg/dL (normal range 2.37–20.7 mg/dL), a free lambda of 10.60 mg/dL (normal range 4.23–27.7 mg/dL), and an IgA kappa in the urine. The serum protein electrophoresis was normal, although immunofixation revealed a small IgM peak.

The patient had no neurological symptoms, and clinical examination did not reveal a peripheral neuropathy nor were there skin lesions. An autoantibody screening was negative for a rheumatological disease.

In view of the deteriorating full blood count, an evaluation of the bone marrow was performed. The bone marrow aspirate resulted in a “dry tap”; however, the bone marrow biopsy showed an extensive infiltration by lymphoid cells with scarce eosinophilic cytoplasm and large nucleoli and being located predominantly in the vessels of the bone marrow. These lymphoid cells stained positive for CD20 and negative for CD3, CD19, CD30, CD117, CD34, CD138, CD68, myeloperoxidase, and ALK. There was no increased presence of reticulin or evidence of fibrosis; CD79A and CD79B were not used in the immunohistochemical analysis. Immunohistochemistry showed a positive result for Bcl-2, although the antibody against MUM1 was not used. Serum antibodies against hepatitis B, hepatitis C, and CMV were negative. Serum antibodies against EBV were positive both for IgM and IgG suggesting a previous but not a new infection. As such, an evaluation of the lymphoma cells for the expression of EBV using immunohistochemical or in situ hybridization was not carried out.

**Figure 1:**
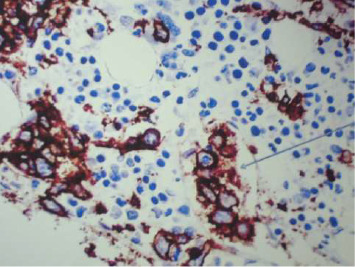
CD20-positive large B-cells diffusely infiltrating the bone marrow and especially located in the intravascular compartment (blue arrow).

**Figure 2:**
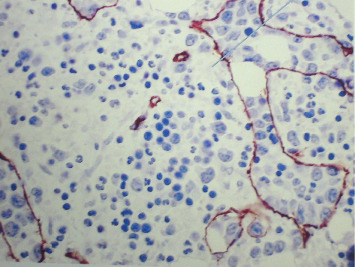
CD34-negative large B-cells within the blood vessels (blue arrow); vascular endothelia positive for CD34 (brown staining).

**Figure 3:**
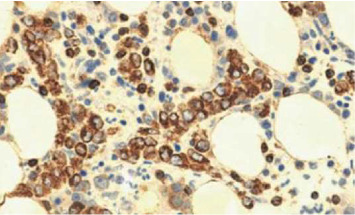
The expression of Bcl-2 in intravascular large B-cell lymphoma.

The findings were consistent with intravascular large B-cell non-Hodgkin's lymphoma (Figures 1, 2, and 3).

Analysis of the CSF using cytology and flow cytometry did not reveal the presence of lymphoma cells.

At the time of the initial diagnosis of intravascular lymphoma, there was a deteriorating full blood count, the levels of triglycerides was 831 mg/dL (normal range 0–150 mg/dL), the LDH 816 U/L (normal range 0–232 U/L), a ferritin of 3120 ng/mL (normal range 30–400 ng/mL), a pancytopenia, fibrinogen level of 302 mg/dL (normal range 200–400 mg/dL), splenomegaly without adenopathy, and fever greater than 38.5°C, without an infective cause being detected. The bone marrow did not demonstrate hemophagocytosi. In Chile, the measurement of the Interleukin 2 receptor or the presence of a low or absent nature killer cell activity is not available.

The patient was treated with R-CHOP (rituximab, cyclophosphamide, doxorubicin, vincristine, and prednisone), achieving a complete remission after completing six cycles of treatment. A bone marrow biopsy showed a hypercellular marrow with no evidence of lymphoma. In addition, a positron emission computerized tomography did not reveal hypercaptation in the lymph nodes, spleen, or bone. The patient had a normal full blood count and normal levels of LDH, ferritin, and triglycerides consistent with a complete remission. The endocrine function had also improved no longer requiring testosterone, hydrocortisone, or thyroxine, with the implication that the endocrine failure was secondary to infiltration by lymphoma. After achieving a complete remission, the proteinuria was again in the normal range.

The patient presented 6 months later to the local Emergency Services, feeling increasing unwell, with a 9-day headache, dyspnea, dry cough, and fever up to 387°C. Blood tests were normal except for an elevated LDH of 897 U/L and elevations in the C-reactive protein of 85 mg/L, a D-dimer of 2881 ng/mL, a ferritin of 350 ng/mL (normal range 30–250 ng/mL), and a fibrinogen of 237 (normal range 200–450 mg/dL). A thoracic CT scan showed interstitial infiltrations in both lungs with ground glass areas and pseudo-nodules. Thoracic, abdominal, and pelvic angio-CT did not reveal any thrombosis. With a provisional diagnosis of influenza, even though the respiratory film array was negative, the patient was discharged the same day being treated with antipyretics. The patient consulted the Emergency Service 3 days later with a worsening symptomology, although a thoracic CT showed no changes in the images.

A platelet count revealed a thrombocytopenia of 116,000 × 10^3^/μL and the presence of normoblasts in the peripheral blood, but no schizocytes were observed, and tests for viral infections including influenza and SARS-CoV-2 were negative. The treatment was changed to amoxicillin-clavulanic acid plus acyclovir, and the patient was discharged the same day. The patient's symptoms worsened with increasing dyspnea and as a result the patient returned to the Emergency Services.

By this time, the LDH had risen to 2308 U/L and the D-dimer to 4394 ng/mL. The full blood count showed a leukoerythroblastic anemia with 1 erythroblast/100 white cells and a platelet count of 32,000 × 10^3^/μ/L and progression of the pulmonary infiltration on a thoracic CT. An infection screen for viral, bacterial, and mycotic infections was all negative. The patient was evaluated 14 days later after the initial evaluation by the Emergency Services by the Hematology Department where a relapse of the lymphoma was diagnosed, based on the thoracic CT findings, the highly elevated LDH, leukoerythroblastic anemia, and thrombocytopenia. The deterioration of the full blood count is shown in Table 3.

Based on the hemophagocytic lymphohistiocytosis by the Histocyte Society and update after the HLH-94 study, the patient had fever but no hepatopesplenomegaly was detected, the serum ferritin was less than 500 ng/ml, the fibrinogen level was normal being greater than 150 mg/dl, and the measurement of sCD25 is not available in Chile. There was an increasing pancytopenia but the bone marrow biopsy did not reveal hemophagocytosis with the implication that HLH was not the primary cause, although it has been described in patients with lymphoma (6).

With a rapidly progressive multi-organ failure, the patient was transferred to the Intensive Care Unit. Here the patient lost consciousness, although the cerebrospinal fluid did not show infection or infiltration by lymphoma. The patient underwent mechanical ventilation and dialysis for the pulmonary and renal failure, respectively, with a deteriorating hepatic function. Proteinuria was again elevated as in the initial presentation, even though after remission the levels had returned to normal.

The patient received multiple transfusions of both packed red cells and platelets. At this moment, schistocytes were detected in the blood and a diagnosis of a thrombotic microangiopathy was made in the context of a relapse of the lymphoma; the level of ADAMTS-13 was 33% of normal. Two sessions of plasmapheresis failed to improve the clinical findings; although prednisone at a dose of 2 mg/kg was started, the patient died 2 days later. In view of the rapid deterioration of the patient's clinical situation, further chemotherapy was not considered.

The results of a bone marrow biopsy taken in this time confirmed a relapse of the lymphoma, although the results were only available after the patient's death.

## 3. Discussion

Intravascular large B-cell non-Hodgkin's lymphoma is a rare lymphoma characterized by the predominant, if not exclusive, growth of large cells within the lumen of different sized blood vessels [1]. This case is unusual in that the patient presented with multiple endocrine failure and a “pulmonary embolism.” As mentioned previously, there are three variants of intravascular lymphoma [3]. In the classical variant, two organ-related features are found: firstly multiple endocrine failure, most frequently the pituitary, thyroid, and adrenal glands as was found in this case, and lung involvement which radiologically appears as a ground glass appearance with nodules [4]. In this case, the patient had a thoracic angio-CT showing a filling detect consistent with a pulmonary artery thrombosis but no other changes seen on CT scan. This filling defect, in retrospect, may have been due to lymphoma cells blocking the pulmonary arteries; after establishing the definitive diagnosis, the hypothesis was that this filling defect was due to lymphoma infiltration, although there was no direct evidence of this.

The cutaneous variant has dermatological findings of a heterogeneous nature including violaceous plaques, nodules, large solitary plaques, painful blue-red palpable nodules, ulcerated nodules, and erythematous and desquamated plaques, which were not seen in this case.

The third variant is that of the hemophagocytic syndrome with bone marrow failure, fever, hepatosplenomegaly, and thrombocytopenia in 73%–100% of cases. These findings are not seen in the other two variants. Anemia is seen in 63% of cases, thrombocytopenia in 29%, leukopenia in 24% with an elevated ESR, and a monoclonal serum component in 14% of cases [3]. Bone marrow biopsy showed large B-cells within the sinusoids; only in intravascular B-cell lymphoma are the vessel lumens selectively colonized [5]. Immunohistochemistry showed that these cells expressed CD20 and CD10 but not CD5. In the HLH-94 trial, the diagnosis of the hemophagocytic variant was based on the findings of fever, splenomegaly, bicytopenia, hypertriglyceridemia, and/or hypofibrinogenemia and hemophagocytosis. As a result of the HLH-2004 trial, three additional criteria were added: low/absent NK-cell activity, hyperferritinemia, and a high level of soluble interleukin-2 receptor [5]. To qualify as the hemophagocytic variant, five of these eight criteria must be present, except when there is a family history of hemophagocytosis or a molecular diagnosis consisitent with this variant of intravascular lymphoma being present. The patient when the pancytopenia began fulfilled five of the eight criteria, although in the bone marrow biopsy, hemophagocytosis was not detected. As such, the patient could be classified as having the hemophagocytic variant [5].

However, as reported in this clinical case, the classical variant seems to show a slower disease progression, some 5 months in this case and 8 months in a case report of a 74-year-old man with pulmonary nodules [6]. Although in the published literature, the transformation from the classical variant to the hemophagocytic variant is not documented, it would appear that the hemophagocytic variant is very much more aggressive in its progression. Several biomarkers have been associated with a worse prognosis. The percentage of lymphoma expressing Ki-67 is one such parameter. The higher the Ki-67 proliferation index, the more aggressive the disease. However, different cutoff points have been suggested, of more than 60% [7] and of more than 70% [8]. Other studies of the Ki-67 proliferation index have included all subtypes of large B-cell lymphoma and not specifically intravascular lymphoma. Hasselblom et al. reported that a low Ki-67 proliferation index was associated with a poorer prognosis [9]. However, a more recent study reported that the Ki-67 proliferation index was associated with a poorer prognosis [10].

In this clinical case, the maximum time from the first symptoms to hospitalized was 43 days. The time period between the patient having a normal full blood count to having a severe pancytopenia was 7 days, consistent with an aggressive disease. Similarly, at the time of relapse, the time between a normal full blood count and developing a severe pancytopenia was 8 days, again consistent with an aggressive disease. In this case, the Ki-67 proliferation index was not used; the clinical picture demonstrating an aggressive disease and a high Ki-67 index does not change treatment.

Intrathecal methotrexate or high-dose methotrexate to prevent CNS relapse was not given in this patient. Since the introduction of rituximab to CHOP chemotherapy, the use of methotrexate as prophylaxis is controversial. CNS relapse in all patients with diffuse large B-cell lymphoma has been reported to be between 3% and 5% [11]. Guidelines differ globally for the use of intrathecal prophylaxis, with different levels of evidence to support their recommendations from 2A in the NCCN guidelines to 4C in the ESMO recommendations [12]. Most reports of the use of IT methotrexate have been on patients with diffuse large B-cell non-Hodgkin's lymphoma rather than intravascular lymphoma [12]. There are no randomized controlled trials of the efficacy of CNS prophylaxis in addition to standard R-CHOP chemotherapy. In large retrospective studies of IT high-dose methotrexate, there have been no significant differences in reducing CNS relapse, but it is associated with a significantly higher rate of therapy-related toxicity [13]. Puckrin et al. reported that high-dose methotrexate was ineffective as prophylaxis for CNS relapse [14]. Similarly, Tolley et al. reported that high-dose methotrexate did not prevent CNS relapse [15]. With regard to prophylaxis to prevent CNS relapse using high-dose methotrexate, other studies have reported that it was ineffective [16, 17]. However, another study showed a benefit of using high-dose methotrexate in decreasing the risk of CNS relapse [18].

Detection of the presence of lymphoma cells in the CNS traditionally used cytological analysis of the CSF but is associated with a false negative rate of 20%–60%, and therefore flow cytometry is being currently used [19]. One new method being investigated is the detection of circulating tumor DNA in the CSF to detect and monitor the presence of lymphoma cells in the CNS [20, 21].

Lung infiltration caused by intravascular lymphoma presents as progressive dyspnea, fever, a nonproductive cough, and B type symptoms. It is relatively frequent, reported in approximately 60% of cases but can be interpreted as infection, infarction, or a diffuse interstitial pneumonia [6]. The symptoms are associated with elevated inflammatory parameters such as the C-reactive protein, a very high LDH, and a thoracic CT scan showing bilateral ground glass infiltrations especially in the superior lobes as well as pseudo-nodules [6, 22, 23].

First-line treatment is with R-CHOP, with a reported complete remission rate of 88% and an overall response rate of 91% with an overall 3-year survival of 81% of cases [10, 11]. After 6 cycles of R-CHOP, the patient was in complete remission and without hormonal replacement, suggesting that the multiple endocrine failure was due to infiltration by lymphoma. The first case of reversible hypopituitarism in patients with intravascular lymphoma was reported in 2016. The same authors reported that there were a total of 20 similar cases with hypopituitarism being associated with intravascular lymphoma. However, the changes in the endocrine function during follow-up were described in only two cases. In these two cases, the patients required hormonal replacement therapy [24].

What is important in this clinical case is that at the time of presentation, a relapse of lymphoma was not considered. The symptoms and nonspecific changes on the thoracic CT in association with an elevated LDH as well as the failure of response to standard therapy suggested the possibility of a relapse. A decreased platelet count at the second evaluation should have reaffirmed the possibility of a relapse. At the third evaluation, the leukoerythroblastic anemia implied a relapse of the lymphoma, albeit in this short period of time. The symptoms of the patient, including a constant headache of 9 days, have been previously reported even in the absence of neurological signs, a normal cerebral CT, and lumbar puncture, as seen in this case [25].

The absence of an infective cause for the symptoms again suggested the possibility of relapse. Intravascular lymphoma has been reported to be associated with multiple organ failure due to infiltration [3]. However, the detection of schistocytes in the peripheral blood film and multi-systemic organ failure was more consistent with a thrombotic angiopathy. The level of ADAMTS-13 was decreased, at 33% of normal. To distinguish between TTP and other thrombotic microangiopathies, clinical findings or a scoring system, such as the PLASMIC score or the French TMA score, has been used [26, 27]. Furthermore, there is no consensus about the cutoff value of ADAMTS-13 to support a diagnosis of thrombotic thrombocytopenic purpura. An American guideline suggests cutoff point of 70% while a European report suggest a cutoff point of 40%. A 10% level is somewhat arbitrary, and more recent guidelines by the International Society on Thrombosis and Haemostasis suggest that a level of 10%–20% of ADAMTS-13 is potentially equivocal [28]. It is important to differentiate cancer-induced TTP from other causes as plasmapheresis is largely ineffective in patients with cancer, as in this case report there was only a partial response.

Interestingly, Vandermeersch et al. [29] presented a case where a patient with IVLCBL presented as central nervous system pseudo-vasculitis: a rare diagnostic challenge. In another clinical case, it was reported that an occult IVLCBL presented as postoperative thrombotic microangiopathy [30].

The limitation of this of this case report is that it was not possible to evaluate the molecular and/or immunophenotype at the time of diagnosis and relapse to determine if there was clonal evolution of the lymphoma.

## 4. Conclusions

This case report is interesting in several ways; intravascular lymphoma presents with a variety of nonspecific symptoms and clinical and findings. As it is an infrequent subtype of large B-cell lymphoma, the majority of publications are case reports and a small series of cases. In this case, the presence of splenomegaly and an elevated LDH suggested the possibility of a lymphoproliferative disease. With the development of bone marrow failure, a bone marrow biopsy confirmed the diagnosis of IVLCBL. Retrospectively, this definitive diagnosis explained the findings as being consistent with the classical variant of intravascular lymphoma. As previously reported in a case study, this variant has a relatively slow progression, with a time period of several months. The transformation to the hemophagocytic variant showed an aggressive disease with rapidly progressing bone marrow failure. This, to the best of our knowledge, has not been previously described. Complete remission was achieved using immunochemotherapy and was demonstrated by the PET-CT and bone marrow evaluation. The patient then presented with respiratory symptoms, initially diagnosed as influenza, although an elevated LDH and 3 days later a mild thrombocytopenia should have suggested a relapse. With this new clinical presentation, the respiratory system not having previously involved highlights the different forms that intravascular lymphoma may present. Multi-organ failure has been previously described as a result of infiltration by the lymphoma. However, the presence of a thrombotic microangiopathy resembling TTP has been described in one case report. Plasmapheresis is not effective in these few cases, and the ADAMTS-13 was not consistent with classical TTP. In summary, intravascular lymphoma presents with a myriad of nonspecific findings and requires a multidisciplinary approach to confirm the diagnosis and the appropriate management.

## Figures and Tables

**Table 1:** Endocrine function before immunochemotherapy and when in remission.

Hormone	Normal range	Pretreatment	In remission
TSH (IU/mL)	1.85–3.85	5.45	1.90
Free thyroxine	0.90–1.75	0.49	1.53
Free testosterone (ng/dL)	284–800	47	351
8 a.m. cortisol (mcg/dL)	6.2–19.4	7.0	18.6
ACTH (pmol/L)	2–11	2.8	3.2
Prolactin (ng/mL)	4.6–21.4	40.0	18.1
FSH (IU/mL)	1.5–12.4	3.5	3.6
LH (mIU/mL)	1.7–8.6	3.1	3.2

**Table 2:** Evolution of the full blood count pretreatment.

Parameter	Normal range	September 2022	November 8, 2022	November 15, 2022	November 23, 2022
Hemoglobin (gr/dL)	13.0–18.0	15.1	11.4	9.1	7.4
White cell count × 10^3^/μL	4500–12,000	8000	5200	3500	3750
Absolute neutrophil count × 10^3^/μL	1800–6000	4300	3472	2380	2500
Platelet count 10^3^/μL	150,000–400,000	273,000	97,000	81,000	35,000
Erythroblasts/100 leukocytes	0	0	0	2	216
Schizocytes	0	0	0	0	0

**Table 3:** Evolution of the full blood count from the time of complete remission and during relapse.

Parameter	Normal range	May 2023 in remission	October 3, 2023, relapse	October 14, 2023	November 3 (pretransfusion)
Hemoglobin (gr/dL)	13.0–18.0	15.1	11.1	9.1	7.4
White cell count × 10^3^/μL	4500–12,000	8000	5200	3500	3750
Absolute neutrophil count × 10^3^/μL	1800–6000	4300	3372	1300	700
Platelet count × 10^3^/μL	150,000–400,000	273,000	97,000	81,000	35,000
Erythroblasts/100 leukocytes	0	0	1	7	21
Schizocytes	Negative	Negative	Negative	Negative	Positive
